# Investigation of shear-induced rearrangement of carbon nanotube bundles using Taylor–Couette flow†

**DOI:** 10.1039/d1ra07354k

**Published:** 2021-11-26

**Authors:** Haemin Lee, Jinhwan Park, Hyunjung Cho, Jaegeun Lee, Kun-Hong Lee

**Affiliations:** Department of Chemical Engineering, Pohang University of Science and Technology 77 Cheongam-Ro, Nam-gu Pohang Gyeongbuk 37673 Republic of Korea ce20047@postech.ac.kr +82-54-279-2003; LG Chem R&D Campus Daejeon 188 Munji-ro, Yuseong-gu Daejeon 34122 South Korea; School of Chemical Engineering, Pusan National University 2 Busandaehak-ro 63 Beon-gil, Geumjeong-gu Busan 46241 Republic of Korea jglee@pusan.ac.kr +82-51-510-2495

## Abstract

Macroscopic assemblies of carbon nanotubes (CNTs) usually have a poor alignment and a low packing density due to their hierarchical structure. To realize the inherent properties of CNTs at the macroscopic scale, the CNT assemblies should have a highly aligned and densified structure. Shear-aligning processes are commonly employed for this purpose. This work investigates how shear flows affect the rearrangement of CNT bundles in macroscopic assemblies. We propose that buckling behavior of CNT bundles in a shear flow causes the poor alignment of CNT bundles and a low packing density of CNT assemblies; the flow pattern and the magnitude of shear stress induced by the flow are key factors to regulate this buckling behavior. To obtain CNT assemblies with a high packing density, the CNTs should undergo a laminar flow that has a sufficiently low shear stress. Understanding the effect of shear flow on the structure of CNT bundles may guide improvement of fabrication strategies.

## Abstract 2

The rearrangement of CNT bundles depends on the flow pattern and flow-induced shear stress. When the Taylor–Couette flow is stable and laminar, and has sufficiently low shear stress, CNT assemblies assume a highly aligned and densified structure.

## Graphical abstract

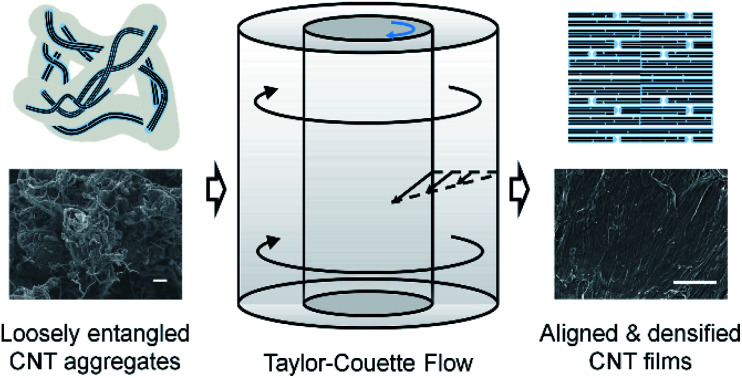

## 1. Introduction

**Fig. 1:**
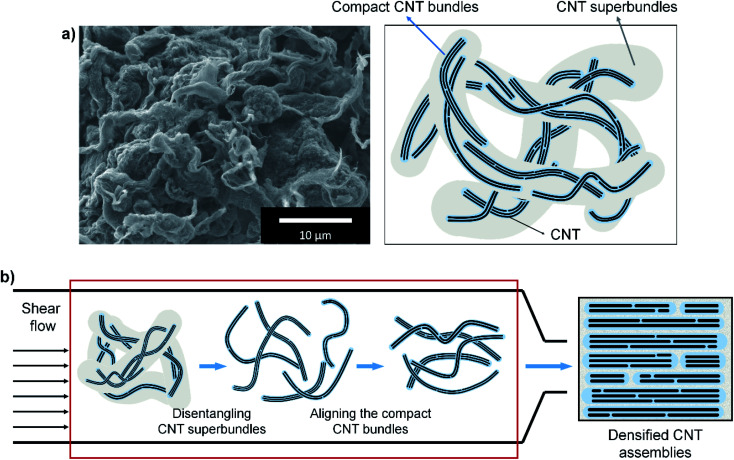
(a) SEM image of a CNT assembly (Left) and schematic image of hierarchical structure (Right). (b) Strategy to fabricate highly-densified CNT assemblies by exploiting shear flow. Loosely entangled CNT superbundles disentangle into compact CNT bundles, which rotated and become aligned in the shear flow. Extrusion of these compact CNT bundles can yield CNT assemblies that have a high packing density. Shaded areas indicate CNT bundles (blue: compact bundles, gray: superbundles).

Carbon nanotubes (CNTs) can form macroscopic assemblies like CNT fibers or films, which typically have hierarchical structures (Fig. 1a).^1–4^ Individual CNTs form compact CNT bundles, in which adjacent CNTs strongly attract each other by strong van der Waals (vdW) forces due to their closeness;^1,2,5–7^ these compact bundles seem to have a highly densified structure.^1^ The compact bundles are loosely entangled by weak vdW force and form large CNT superbundles.^8,9^ These superbundles are physically entangled and attract each other weakly in CNT fibers and films.^1,3,9,10^ As a result, numerous voids of micrometer scale and smaller, inevitably form in CNT assemblies.^1,3,9,10^

The properties of CNT fibers and films depend significantly on their packing density. The mechanical strength relies dominantly on the inter-tube frictions, and electrical conductivity is dependent on the contact area between CNTs and the number of contacts.^1,4,11–15^ Therefore, to fabricate strong and highly conductive CNT fibers and films, they should be perfectly densified at and below the micrometer scale. To achieve high packing density, an aligned structure of CNTs is most desirable.^4,16^ Theoretical studies predict that the properties of CNT fibers could be comparable to those of individual CNTs, if the CNT fiber is composed of sufficiently long CNTs (length/diameter > 10^5^) and is perfectly densified.^4,17^

Shear-aligning methods are a promising approach to obtain fibers and films that have highly aligned and densified structure.^18^ Flow-induced shear stress arranges particles in the flow direction. The methods are applicable to various systems regardless of their chemistry,^19–21^ and are advantageous in mass production; for example, the method of extrusion has been widely used in industry.^22^

Most studies that reported on shear-aligning methods have tried to disperse CNTs individually before aligning by functionalizing CNTs or using surfactants.^23–26^ Damages induced by functionalization and residual surfactants might decrease the mechanical properties and the electrical conductivity of CNT assemblies.^27,28^ Using super-acid or polyelectrolyte solutions, it is possible to disperse CNTs without functionalization.^6,7,27–30^ However, these methods are only applicable to CNTs with high crystallinity, and are difficult to handle because they use troublesome reagents (*e.g.*, super-acid or sodium).^4,6,16,27,28,30^ Several studies improved the alignment of CNTs by applying shear force directly to CNT assemblies without dispersing them,^1,31^ but small-scale voids were not effectively removed.

We expect that highly aligned and densified assemblies of CNTs can be obtained by aligning and densifying these compact CNT bundles without debundling the compact bundles (Fig. 1b), because the compact CNT bundles seem to have a highly aligned and densified structure. The loosely entangled CNT bundles can be easily disentangled into compact CNT bundles in shear flow without introducing damage or using additional materials.^32^ We tried to disentangle and align loosely entangled CNT bundles by using shear flow. In order to effectively control the structure of CNT assemblies, it is necessary to clearly understand the effect of shear flow on the disentangling and aligning of bundles.

Here, we investigate how shear flows affect the rearrangement of CNT bundles by considering disentanglement and alignment of them. We used a Taylor–Couette (TC) reactor to develop various flows. The TC reactor consists of two concentric cylinders and can control the flow pattern and the magnitude of shear stress.^19,33–36^ We assessed the effect of shear flows by observing the morphology of CNT buckypapers that had been subjected to flows with different condition. Unstable flow always led to highly entangled structures, whereas laminar flow could generate either entangled or highly densified structures, depending on the shear stress induced by flows. When laminar flow has sufficiently high shear stress, the obtained CNT bundles were highly entangled, which was counterintuitive. We propose that buckling behavior of CNT bundles causes the poor alignment of CNT bundles and the low packing density of CNT assemblies, and that this behavior is dependent on both the flow pattern and the shear stress. Our results can help to increase understanding of the mechanisms of change in the microstructure of CNT materials and may guide development of methods to fabricate CNT materials that have desired structures.

## 2. Materials and methods

### 2.1. Materials

We used single-walled CNTs (Zeon Nano Technology Co., Ltd., Japan) that have diameter of 3–5 nm and length >100 μm. They were mixed with solvents (butyl benzoate or benzyl benzoate) purchased from Sigma-Aldrich. The solvents were chosen by considering the Hansen solubility parameter and the viscosity.

### 2.2. Formation of CNT buckypapers under various flow conditions

A customized TC reactor was used to develop various flows selectively. This reactor consists of two concentric cylinders, separated by a gap of 2 mm (outer radius *R*_out_: 42 mm, inner radius *R*_in_: 40 mm). The cylinders are made of Teflon and can rotate individually; the rotation rate *ω*_in_ of the inner cylinder was controlled up to 1500 rpm (corresponds to an apparent shear rate γ̇ = 3000 s^−1^), while the outer cylinder was fixed stationary (*ω*_out_ = 0 rpm).

The CNTs were mixed with the solvents and stirred at 300 rpm for >1 day. The mixture of CNTs and solvent was introduced into the gap between the cylinders, and the inner cylinder was rotated. After TC flow mixing, the CNT buckypapers were fabricated by vacuum filtration, then rinsed sequentially with ethanol and deionized water to remove residual solvent and dried in a vacuum oven at 100 °C.

### 2.3. Characterization

The CNT suspensions developed in the TC reactor were characterized using an optical microscope (OM) (BX53F, OLYMPUS), an ultraviolet-visible-near infrared (UV-vis-nIR) spectroscope (S-3100, Scinco), the zeta-potential (ELSZ-100-, Otsuka Electronics), and a viscometer (DHR-1, TA Instruments). In OM measurements, the thickness of the CNT suspensions was fixed at 0.2 mm. To observe the UV-vis-nIR absorbance, the CNT suspensions were loaded in a quartz cuvette with a 1 cm path length and sealed with a Teflon stopper. A scanning electron microscope (SEM, XL30S FEG, FEI), Raman spectroscopy (LabRam Aramis, Horiba Jobin Yvon), and Fourier-transform infrared (FT-IR) spectrometer (Nicolet iS50, Thermo Scientific) were used to characterize the CNT buckypapers.

## 3. Results and discussion

### 3.1. The control of flow pattern and magnitude of shear stress using the Taylor–Couette reactor

Our strategy to study the effect of shear flow is to observe the variation in the average thickness and the alignment of CNT bundles subjected to various flow conditions. For this purpose, we used a TC reactor because it enables control of the flow pattern and the magnitude of the shear stress by changing the rotational speed of each cylinder, and the viscosity of fluids.^19,33–36^

**Fig. 2:**
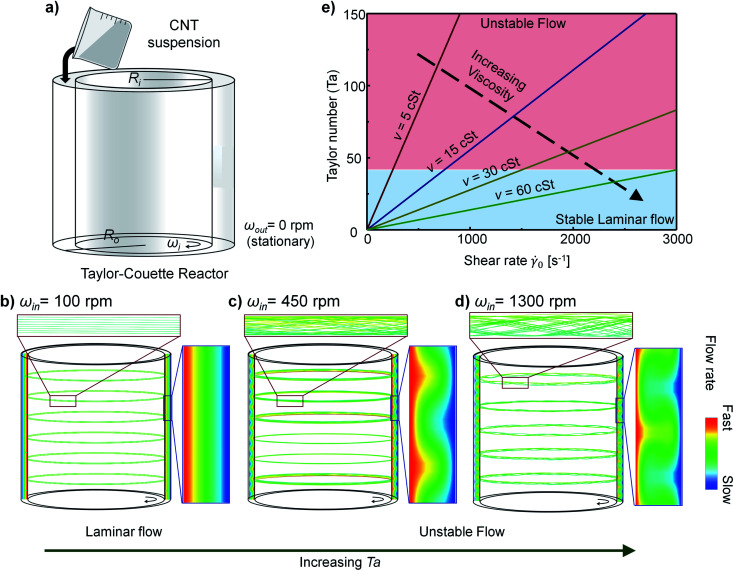
(a) Schematic of the Taylor–Couette (TC) reactor. (b–d) Simulated pattern of TC flows at: (b) *ω*_in_ = 100 rpm, (c) *ω*_in_ = 450 rpm, (d) *ω*_in_ = 1300 rpm; insets: the stream line of each flow. (e) Relationship between *Ta* and characteristic shear rate γ̇_0_ = γ̇(*r* = *R*_in_) of TC flow. The γ̇_0_ at which TC flow remains stable increases with increase in the viscosity of suspension.

The pattern of TC flows could be inferred from the Taylor number *Ta*.^37^ If the outer cylinder is fixed stationary as in our system (Fig. 2a), then1
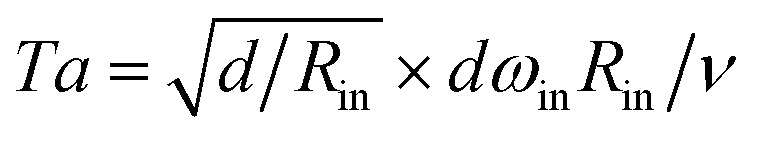
where *d* = *R*_out_ − *R*_in_ is the gap between concentric cylinders, *ω*_in_ is the rotational speed of the inner cylinder, and *ν* is the kinematic viscosity of fluids. Flow is stable and laminar at *Ta* < ∼41.2 (Fig. 2b) but vortex flow or turbulent flow occur at *Ta* > 41.2, and the instability of the flow increases as *Ta* increases (Fig. 2c and d).

The flow-induced shear stress is *τ* = γ̇*η*, where *η* is the dynamic viscosity of the suspension. γ̇ is defined as^38^2
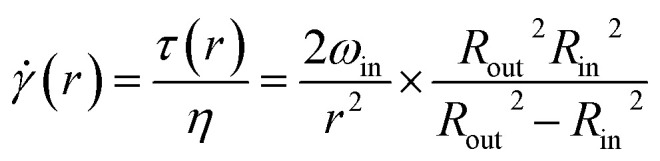
where *r* means the distance from the center of concentric cylinders. According to eqn (2), both *τ* and γ̇ increase as the particle approaches the surface of the inner cylinder (*r* → *R*_in_). Particles experience different shear stress depending on their *r*; we chose *τ*_0_ = *τ*(*r* = *R*_in_) as characteristic shear stress and γ̇_0_ = γ̇(*r* = *R*_in_) as characteristic shear rate to represent the flow conditions of the system.

The relationship between *Ta* and γ̇_0_ was calculated from eqn (1) and (2) by substituting appropriate *ν* and *ω*_in_ (Fig. 2e). As the *ν* of the suspension increased, the flow can remain stable at a substantially high γ̇_0_.

We measured the *ν* of CNT suspensions at various conditions and calculated the *Ta* and *τ*_0_ of flows at each condition (Table 1 and Fig. S1†). Thus, at either laminar or unstable flow regime, flows that induce a wide range of shear stress can be obtained in the TC reactor simply by changing solvents, CNT concentration, and *ω*_in_ (Table 1).

**Table 1:** Taylor number *Ta* and characteristic shear stress *τ*_0_ of Taylor–Couette flows used in this work. Conc: concentration of CNTs; *ω*_in_: rotation rate of the inner cylinder; *τ*_0_: characteristic shear stress *τ*_0_ = *τ*(*r* = *R*_in_) at the surface of the inner cylinder

Conc. [mg mL^−1^]	*ω*_in_ [rpm]	Solvent			
		Butyl benzoate		Benzyl benzoate	
		*Ta*	*τ*_0_ [Pa]	*Ta*	*τ*_0_ [Pa]
1	100	11.5	3.64	4.58	9.21
	450	179	4.76	42.2	20.3
	1300	518	13.7	148	48
3	100	4.47	9.43	2.46	17.2
	450	46.3	18.4	21.1	40.5
	1300	180	39.5	96.3	74.1
5	100	2.24	18.8	1.08	39
	450	22.7	37.7	15.9	53.7
	1300	89.9	79.4	68.8	104
10	100	0.95	44.6	0.24	177
	450	11.0	77.5	4.68	182
	1300	47.7	151	38.5	185

### 3.2. Disentangling the CNT bundles in the shear flow

To improve the efficiency of shear-aligning, loosely entangled superbundles in the CNT aggregates should first be fully disentangled in the flow. Shear flow can disentangle entangled CNT structures when the shear stress induced by flow is larger than the attractive force between CNTs.^39,40^ We observed OM images and the UV-vis-nIR absorbance of the CNT suspensions to assess the disentanglement of CNT bundles at each flow condition.

**Fig. 3:**
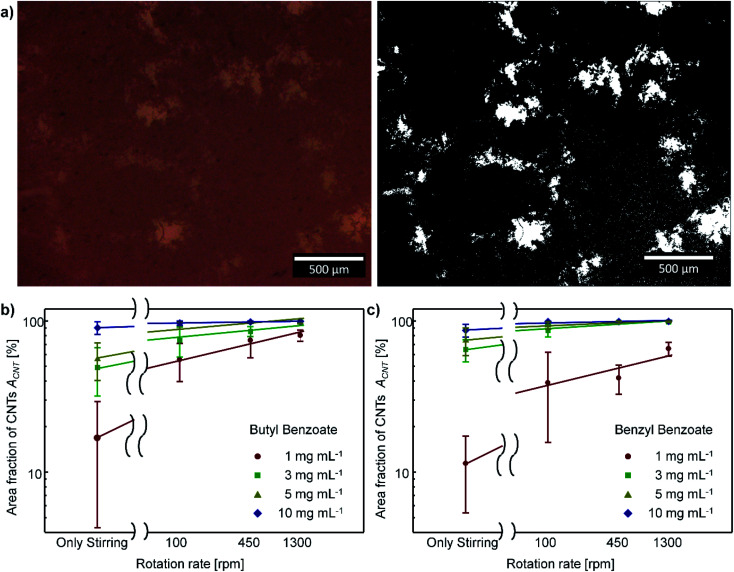
(a) OM images of CNT suspensions (left: raw image, right: after processing). (b) and (c) The area fraction of CNTs in suspension *vs.* rotation rate of inner cylinder *ω*_in_: (b) CNT-butyl benzoate suspension, (c) CNT-benzyl benzoate system. Bars: ±s. d., *n* > 20.

The level of disentanglement of CNT bundles can be qualitatively observed using SEM, TEM, and OM.^41–47^ These microscopy images intuitively show the size distribution of CNT bundles.^45–47^ However, CNT bundles in the suspensions would form an entangled mesh network structure at high CNT concentration.^44^ Accordingly, the OM images of CNT suspensions show that CNT bundles form mesh network structures (Fig. 3a left), which complicates the comparison of the disentanglement level of CNT bundles. Thus, a quantitative approach must be employed to assess the degree of disentanglement of CNT bundles.

At a fixed volume fraction of CNTs in the suspension, the total area covered by CNTs in the suspension should increase as the size of CNT bundles decreases. Hence, the size of CNT bundles can be inferred from the area covered by CNTs in the suspension. We can assess the variation of the area covered by CNTs from the area fraction of CNTs *A*_CNT_ in the OM images, and the *A*_CNT_ is defined as the fraction of the area covered by CNTs to the total area. The *A*_CNT_ is obtained from different CNT suspensions by using Image J software (Fig. 3a).

*A*_CNT_ was much higher in the CNT suspension that had been subjected to TC flow than in the suspension prepared by a mere stirring at 300 rpm (Fig. 3b and c). *A*_CNT_ increased as *ω*_in_ increased. This result is consistent with the results of previous studies that reported decrease in the size of particle aggregates with increasing shear rate when flow-induced shear stress is sufficiently strong.^38,48^ We conclude that the shear stress induced by the rotating TC flow is sufficiently strong to disentangle the CNT bundles.

**Fig. 4:**
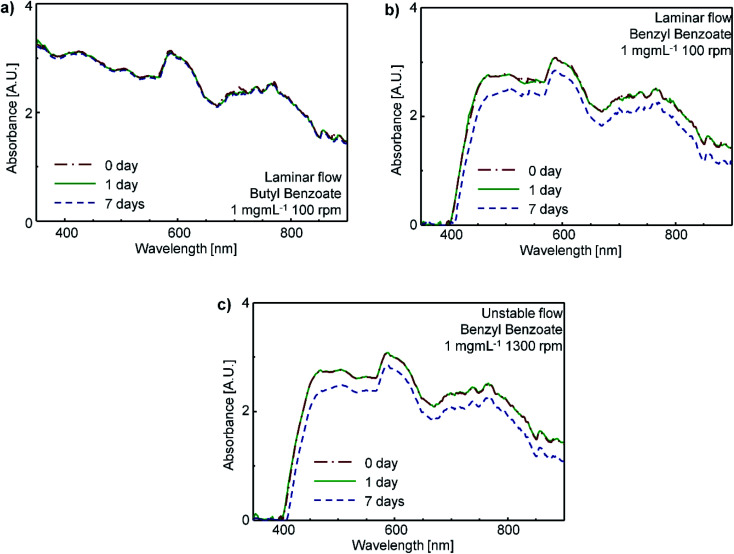
Absorbance of CNT suspensions from as-processed to 7 days. (a) CNT-butyl benzoate suspension (concentration: 1 mg mL^−1^, *ω*_in_: 100 rpm), (b) CNT-benzyl benzoate suspension (concentration: 1 mg mL^−1^, *ω*_in_: 100 rpm), and (c) CNT-benzyl benzoate suspension (concentration: 1 mg mL^−1^, *ω*_in_: 1300 rpm).

To test the stability of CNT suspensions, the UV-vis-nIR absorption spectra of the CNT suspensions were obtained on days 0, 1 and 7 after treatment. These spectra changed little regardless of the flow characteristics (Fig. 4); this result indicates a high stability of the disentangled bundles in the suspensions.

The UV-vis-nIR absorption spectra also provide information about the level of disentanglement of CNTs in the suspension. If SWCNTs are individually dispersed in the suspensions, the UV-vis-nIR absorbance of CNT suspensions should show multiple sharp peaks due to van Hove's singularity, whereas when the CNTs exist as bundles in the suspension, the absorption peaks should be broad.^49^ The CNT suspensions after the TC mixing showed broad absorption peaks at the vis-nIR region (Fig. 4), which indicates that most CNTs in the suspension retained the bundled structures after the TC mixing. Hence, we conclude that the shear stresses induced by the TC flow are not strong enough to completely disintegrate the CNT bundles and disperse CNT individually.

To summarize, the flow-induced shear stress is strong enough to disintegrate large, loosely-entangled superbundles of CNTs to compact CNT bundles, but not strong enough to disintegrate the compact CNT bundles to individual CNTs. This limitation indicates that the interaction force within a compact CNT bundle exceeds the magnitude of effective shear stress defined as the difference between shear stress acting on CNTs within a bundle. The effective shear stress that acts on a CNT bundle should depend on its size (Fig. S2†). The large superbundles are loosely entangled so they will experience stronger effective shear stress and be more apt to disintegrate easily than the small compact bundles (ESI†).

Although the compact CNT bundles do not disintegrate in the shear flow, they assume a highly aligned and densified structure.^1^ Extrusion of these compact CNT bundles may enable production of a CNT assembly that has a highly aligned and densified structure (Fig. 1b).

### 3.3. Aligning the CNT bundles in the shear flow

**Fig. 5:**
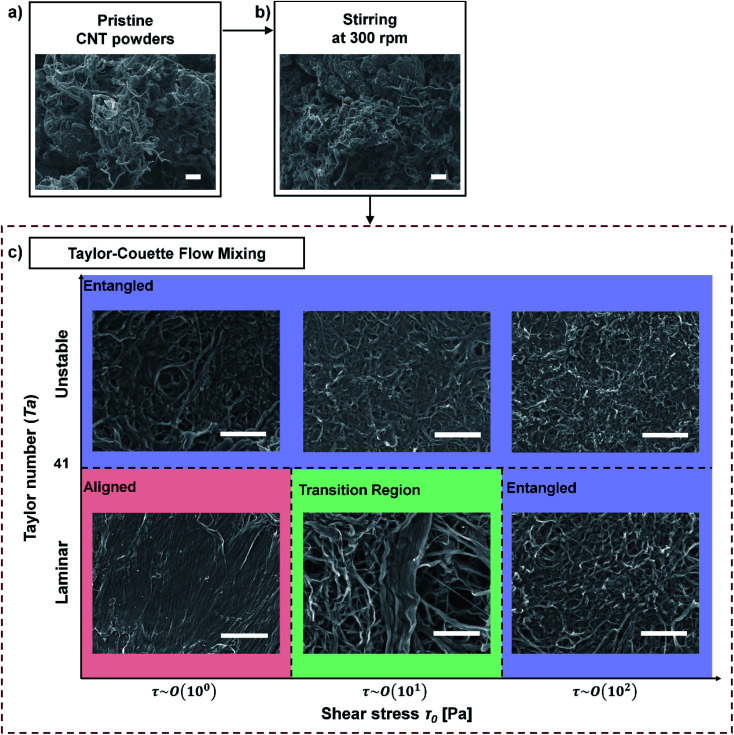
SEM images of pristine CNT powders and CNT buckypapers at processing steps: (a) pristine CNT powder, (b) after stirring at 300 rpm, and (c) after TC mixing. Scale bars: 10 μm.

We tried to identify how shear flow affects the alignment of CNT bundles. Direct measurement is impractical,^40^ so we observed the morphology of buckypapers produced by vacuum filtration of various CNT suspensions (Fig. 5). Pristine CNTs and buckypapers that had been subjected to stirring at 300 rpm had rough surfaces that bore huge aggregated superbundles (Fig. 5a and b), whereas buckypapers that had been subjected to TC flows had a flat and dense structure with few big aggregated bundles (Fig. 5c). These results show that TC flow can effectively disentangle huge CNT aggregates; this conclusion is consistent with OM images.

We confirmed that TC flow did not cause a significant change in the intrinsic properties of CNTs. The Raman spectroscopy and the FT-IR spectroscopy show that the shear stress induced by the TC flow does not cause a meaningful change in the defect concentration and functional group on the CNT surface (Fig. S3†). The zeta-potential *ζ* of CNT suspensions had a negligibly small value (ζ ∼ 0 mV), which indicates that the shear aligning process does not change the surface charge of CNTs.

To understand in detail how the characteristics of TC flow affect the alignment of CNT bundles in buckypapers that were obtained after TC flow mixing, we categorized the SEM images of the papers according to their *Ta* and *τ*_0_.

Interestingly, at *Ta* < 41 (laminar flow), the CNT buckypapers can have either the entangled structure or the highly-densified structure, depending on the *τ*_0_ (Fig. 5c). The CNT assembly was dense and aligned structure when *τ*_0_ ∼ 10^0^ Pa, but highly entangled at *τ*_0_ ∼ 10^2^ Pa. This result is counterintuitive, so we tried to understand why it happened.

We suggest that it is a result of shear flow causing buckling behavior of CNT bundles. Rod-like particles rotate even in laminar flow, and during the rotation, they can buckle.^50–54^ This buckling causes poor alignment of rod particles, and yields a macroscopic assembly that has an undensified structure.^20^ This buckling behavior is controlled by the interplay of the elastic bending force and shear stresses.^50,55–58^

If the shear stress induced by flow is below the threshold stress *τ*_crit_ for buckling, the rod particles rotate in the flow without any deformation in their shape,^50,55^ but at shear stress > *τ*_crit_, the shear flow drives the structural instability during the rotation.^50,59^ According to Euler buckling theory:^50,57,59–61^

3 *τ*_crit_ ∼ (EI)/(*a*^2^*b*^2^)

where EI is bending rigidity of rod particles, *a* is the length of particle, and *b* is the thickness of particles. In theory, EI should grow as the fourth power of the bundle thickness,^62^ so *τ*_crit_ should be proportional to *b*^2^. Thus, as the thickness of a CNT bundle increases, the shear stress required to buckle it also increases.

The structure of CNT bundles in a buckypaper varied with their thickness (Fig. 5c). This result supports our argument that the buckling behavior of CNT bundles induces the poor alignment of CNT bundles. At *τ*_0_ ∼ 10^1^ Pa (transition region in Fig. 5c), aligned and buckled bundles coexist. CNT bundles that have small diameter formed the buckled structure, whereas the thick CNT bundles became aligned. This result indicates that *τ*_crit_ of a CNT bundle increases with increase in its thickness.

At *Ta* > 41 (unstable secondary flow), CNT buckypapers had flat, meshed structures, and the CNT bundles seem to become increasingly wavy as *τ*_0_ increased (Fig. 5). These entangled structures seem to occur due to the vortexes in the flow. Multi-axial shear forces act on the CNT bundles as the direction of local flow constantly changes due to the presence of numerous vortexes in unstable flows.^39,40,44^ The CNT bundles should rotate and buckle in accordance with the flow directions.

**Fig. 6:**
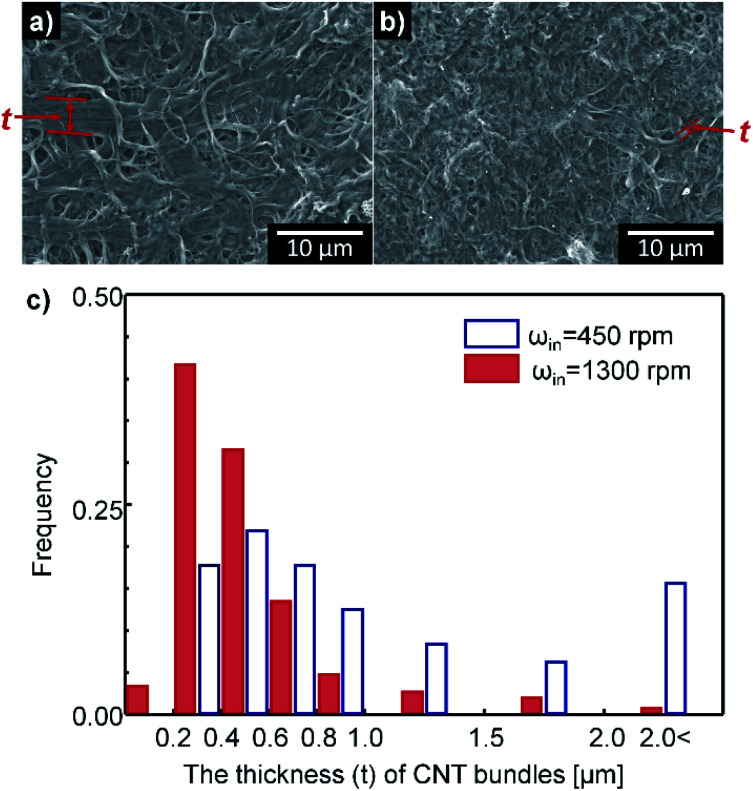
SEM images of CNT buckypaper (benzyl benzoate, 1 mg mL^−1^) at different *ω*_in_: (a) 450 rpm, (b) 1300 rpm; (c) distribution of the thickness *t* of CNT bundles at different *ω*_in_ (empty box: 450 rpm, filled box: 1300 rpm). The average *t* of CNT bundles was 1.3 μm at 450 rpm and 0.51 μm at 1300 rpm.

The disaggregation and morphologies of rod-like particles are affected by the scale of the smallest vortexes.^63–65^ When the particles are larger than the scale of smallest vortexes, the disaggregation and the buckling behaviors are dominant.^65^ The scale of the smallest vortexes can be defined as Kolmogorov's length scale *L*_*k*_, which decreases as the Reynolds number increases (*L*_*k*_ ∼ Re^−3/4^ ∼ (*ω*_in_/*ν*)^−3/4^).^64,65^ Thus, the average thickness of CNT bundles should decrease as *ω*_in_ increases. In agreement with this prediction, CNT bundles subjected to *ω*_in_ = 450 rpm were thicker (avg. *t* = 1.3 μm) than those that had undergone *ω*_in_ = 1300 rpm (avg. *t* = 0.51 μm) (Fig. 6).

To summarize, we propose that the wavy structure of CNT bundles is caused by the buckling behavior of CNT bundles in shear flow, so to obtain highly densified CNT assemblies, these behaviors should be prevented. For this purpose, the CNTs should be subjected to laminar flow with a sufficiently low shear stress.

## 4. Conclusion

We investigated how shear flows affect the rearrangement of CNT bundles by using a TC reactor. We found that the shear stress induced by TC flow could disintegrate loosely-entangled superbundles of CNTs into small, compact CNT bundles, but was not strong enough to disintegrate compact CNT bundles into individual CNTs. We also studied how shear flow aligns CNT bundles. In unstable secondary flow, the CNT bundles bend and become randomly entangled due to vortexes in flow. In laminar flow, interestingly, the alignment of CNT bundles is dependent on the magnitude of the shear stress induced by flow. We propose that the buckling behavior of CNT bundles in a shear flow causes the poor alignment of CNT bundles and a low packing density of CNT assemblies; the flow pattern and the magnitude of shear stress induced by the flow are key factors that regulate this buckling behavior. The understanding on the effect of shear flow can help guide development of methods to fabricate macroscopic CNT assemblies such as CNT fibers, films, and buckypapers with desired microscopic structures.

## Author contributions

Haemin Lee: conceptualization, data curation, formal analysis, investigation, methodology, visualization, writing – original draft, Jinhwan Park: software, visualization, Hyunjung Cho: investigation, methodology, visualization, Jaegeun Lee: conceptualization, formal analysis, methodology, visualization, supervision, writing – review & editing, Kun-Hong Lee: funding acquisition, resource, supervision, writing – review & editing.

## Conflicts of interest

There are no conflicts of interest to declare.

## Supplementary Material

RA-011-D1RA07354K-s001
