# Supplementary material for: Investigation of shear-induced rearrangement of carbon nanotube bundles using Taylor–Couette flow
Source: RSC Adv. 2021 Nov 26;11(60):38152–60. doi: 10.1039/d1ra07354k (PMC9044060; doi:10.1039/d1ra07354k)
Supplement: RA-011-D1RA07354K-s001 [file RA-011-D1RA07354K-s001.pdf]

Electronic Supplementary Material (ESI) for RSC Advances.
This journal is © The Royal Society of Chemistry 2021

Supplementary Information

Investigation of shear-induced rearrangement of carbon nanotube bundles using Taylor-Couette flow

Haemin Lee[1], Jinhwan Park[1], Hyunjung Cho[2], Jaegeun Lee[3,*], and Kun-Hong Lee[1,*]

[1]Department of Chemical Engineering, Pohang University of Science and Technology, 77 Cheongam-Ro, Nam-gu, Pohang, Gyeongbuk, 37673, Republic of Korea

[2]LG Chem R&D Campus Daejeon, 188 Munji-ro, Yuseong-gu, Daejeon, 34122, South Korea

[3]School of Chemical Engineering, Pusan National University, 2 Busandaehak-ro 63 beon-gil, Geumjeong-gu, Busan, 46241, Republic of Korea

*Corresponding authors.

Tel: +82-51-510-2495 E-mail: jglee@pusan.ac.kr (Jaegeun Lee)

Tel: +82-54-279-2003 E-mail: ce20047@postech.ac.kr (Kun-Hong Lee)

1. Simulation of Taylor-Couette flow

3D steady-state numerical simulation was performed to predict the flow in the TC reactor. The governing equations and the geometry followed those of Monfared's work.[1] The TC flow was governed by momentum and continuity equations, and the standard k-ω model was applied. FLUENT software computed the turbulent kinetic energy k and the specific dissipation rate ω. The geometry domain was 'concentric cylinders', and the no-slip boundary condition was imposed on the cylinder walls. The outer cylinder was stationary, and the inner cylinder rotated at rate of ω_{in} up to 1500 rpm. The detailed parameters for the TC reactor are: Inner-cylinder radius R_{in} = 40 mm, outer-cylinder radius R_{out}= 42 mm, height = 100 mm.

We assumed that the solution, which filled the gap between two cylinders, had mass density = 1.1 g cm^{-3} and viscosity = 20 cP. These parameters were randomly chosen in the range of the viscosity and density of CNT suspensions that were used in this work. The simulated results showed that laminar flow occurred when ω_{in} = 100 rpm (Fig. 2b), that unstable secondary flows formed at ω_{in} =450 rpm and ω_{in} = 1300 rpm, and that the flow became increasingly unstable as ω_{in} increased (Figs. 2c, d).

2. Rheological behavior of the carbon nanotube (CNT) suspensions

To predict the flow regime and the magnitude of the shear stress, we observed the rheological behavior of CNT suspensions at a wide range of the CNT concentration and a shear rate (Fig. S1). The viscosity of CNT suspension significantly increased with increasing the CNT concentration. When the concentration of CNTs was sufficiently high, the suspension formed a gel phase (Fig. S1d). At a gel phase, the CNT suspensions were highly condensed and lose mobility. Moreover, since the CNTs formed a highly entangled structure at a gel phase, we predicted that it is difficult to improve the alignment of CNTs efficiently.

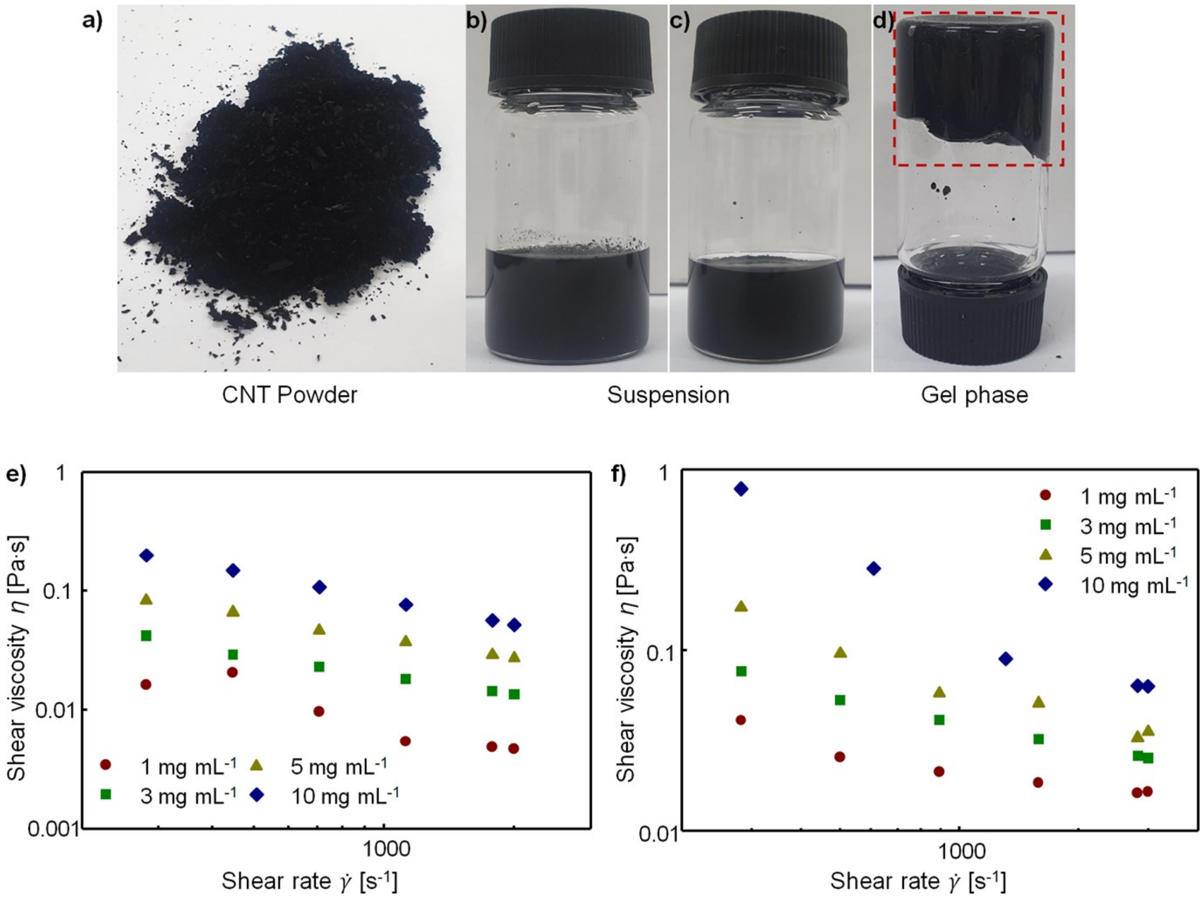

Figure S1. a) The digital image of CNT powders, b-d) Digital images of CNT/benzyl benzoate suspensions with increasing the CNT concentration. b) 3 mg mL^{-1}, c) 5 mg mL^{-1}, and d) 10 mg mL^{-1}. Rheological behavior of the CNT suspensions at different concentration and the shear rate. e) CNT/Butyl benzoate, f) CNT/Benzyl benzoate.

Meanwhile, the viscosity of the CNT suspensions decreases asymptotically as the shear rate increases (Figs. S1e, f). These shear-thinning behaviors indicated that CNTs aligned in the direction of the flow. In addition, the variation in the viscosity was less relevant at a high shear rate (over 1000 s^{-1}, which corresponds to 445 rpm in our system). This result implied that the inertia of fluids was sufficiently high to restrict the tumbling of CNTs in the shear flow.

3. The effective shear force acting on the CNT bundles

Due to the hierarchical nature of CNT structures (Fig. S2a), the disintegration of CNT structures seems to vary with their scale. To disentangle the CNT bundles in shear flow, the shear stress acting on the CNT bundles should be stronger than the attraction force between CNTs or CNT bundles.[2-4]

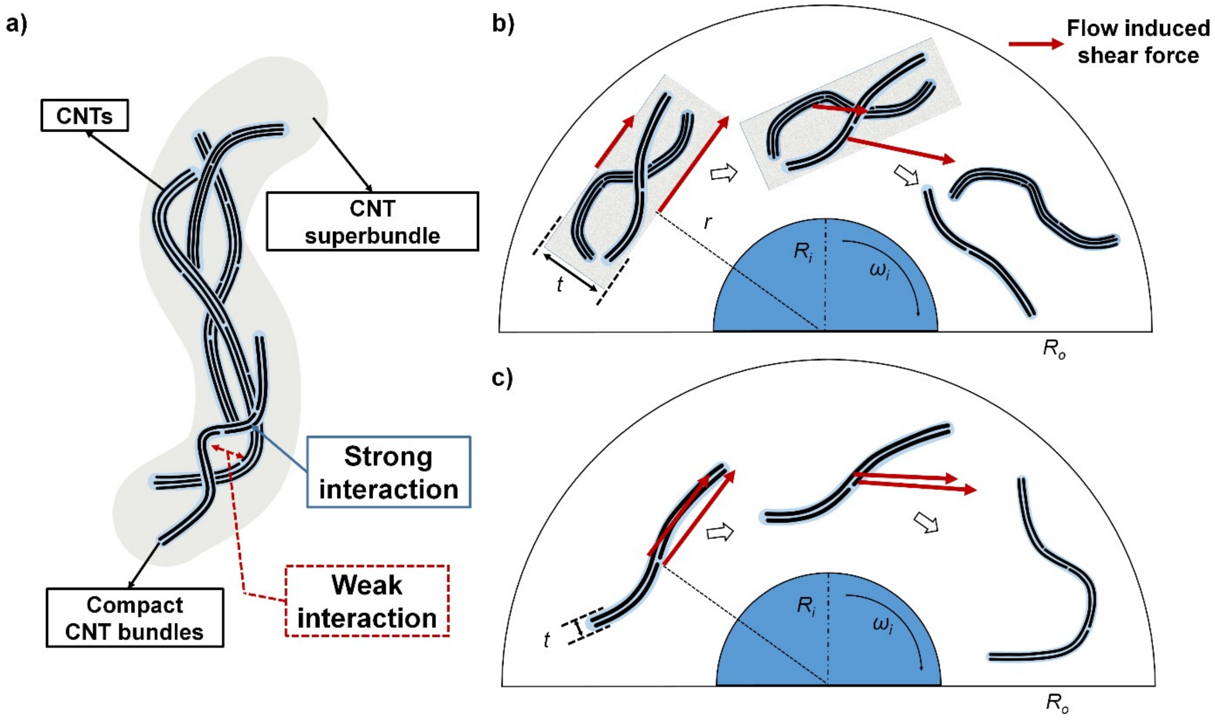

Figure S2. a) The hierarchical structure of CNTs. b) Disentanglement of a CNT superbundle in Taylor-Couette flow c) Disentanglement of a compact CNT bundle in Taylor-Couette flow

When the CNT aggregates are exposed in the shear flow, each CNT or CNT bundle constituting the CNT aggregates will be independently affected. The difference between the shear stresses acting on each CNT bundle can be defined as the effective shear stress for disentangling the CNT bundles. We predicted that the effective shear stress acting on a bundle could be expressed as,[5]

$$\Delta\tau = -\frac{2\eta(\Omega_i)R_{out}^2R_{in}^2}{R_{out}^2 - R_{in}^2}\left[\frac{1}{r^2} - \frac{1}{(r+t)^2}\right] \sim -\tau_0 R_{in}^2 \times \frac{t}{r^3}, \quad \text{(S1)}$$

where r is the distance from the center of concentric cylinders, t is the thickness of CNT bundles, the $\tau_0 = \tau(r = R_{in})$ is characteristic shear stress, ω_{in} is the rotational speed of the inner cylinder, and η is the kinematic viscosity of fluids. According to Equation S1, the effective shear forces acting on the CNT bundles is proportional to the thickness (t) of CNT bundles.

In the case of disintegration of a CNT superbundle (Fig. S2b), as its thickness is generally micrometer scale,[6] the $\Delta\tau$ seems to be higher than several tens mPa. The optical microscopy (OM) and scanning electron microscopy (SEM) measurements showed that the CNT bundles were disintegrated in the Taylor-Couette flow (Figs. 3, 6), which indicates that the $\Delta\tau$ is sufficiently large to untangle the CNT superbundle.

Meanwhile, in the case of a compact CNT bundle (Fig. S2c), the distance between CNTs is only a few nanometers (mostly 1~2 nm),[6] the $\Delta\tau$ is less than 1 mPa; this is much weaker than the vdW attractive forces between CNTs. As a result, the shear stress induced by the Taylor-Couette flow is inadequate to disperse CNTs individually; this result corresponds to the UV-vis-nIR measurements.

To summarize, the flow-induced shear stress is strong enough to disintegrate the CNT superbundles into compact CNT bundles, but not strong enough to disintegrate the compact CNT bundles into individual CNTs.

4. The effect of shear stress induced the TC flow on the defects on the CNT surface

The alignment of CNTs may be influenced by the defect concentrations of CNTs and the degree of the functionalization on the CNT surface as well as the condition of flows. We should consider the effect of other factors like the defect concentration, amount of the functional group, and surface charge, etc. Raman spectroscopy, Fourier-transform infrared (FT-IR) spectroscopy, and zeta-potential ζ measurements were used to investigate the changes in these factors during the TC flow mixing.

I_G/I_D ratios of Raman spectra were very similar; it ranged from 3 to 4 (Fig. S3a), indicating that the processing under TC flows changes the CNT alignment without significantly increasing defect concentrations.

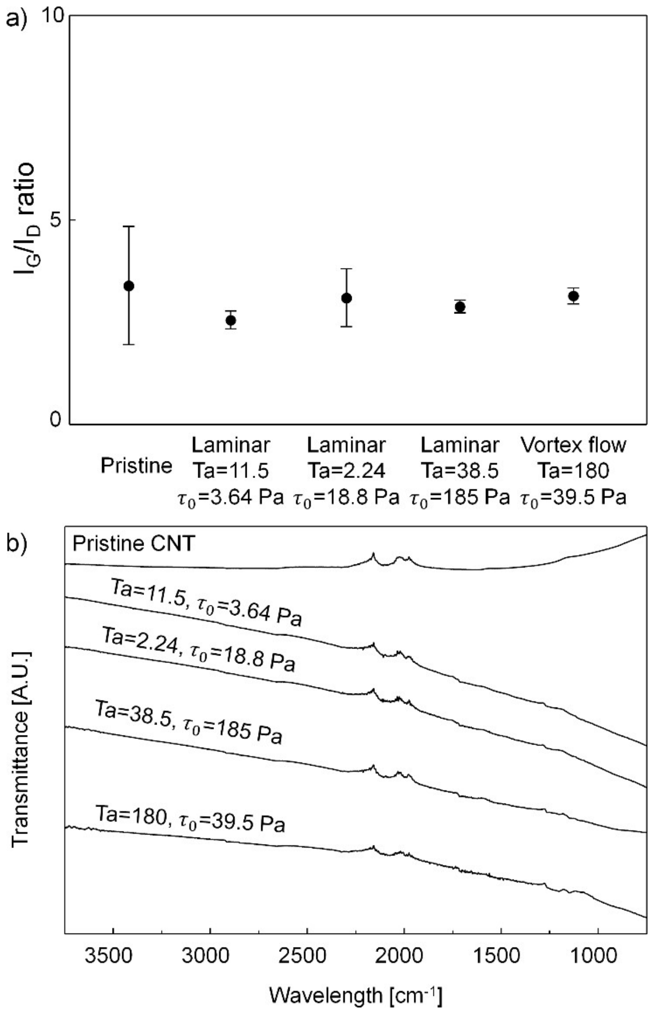

Figure S3. a) I_G/I_D ratio of Raman spectroscopy and b) FT-IR spectra of the pristine CNT and CNT buckypapers that are subjected the Taylor-Couette flow with different condition

FT-IR spectra of pristine CNTs and the CNT buckypapers that are subjected to different TC flows are very similar and only show peaks near 1900 ~ 2200 cm^{-1} which are ascribed to carbon structures (Fig. S3b). This agreement implies that no functional groups were formed during the TC flow mixing.

In addition, the zeta-potential of CNT suspensions had a very small value ($\zeta \sim 0$ mV), which indicates that the shear aligning process does not change the surface charge of CNTs. This result is consistent with the I_G/I_D ratio and FT-IR spectra.

Raman spectroscopy, FT-IR spectra, and the zeta-potential measurements show that the shear stress induced by Taylor-Couette flows did not introduce defects and functional groups during disentangling and aligning the CNT bundles. We concluded that the conditions of the flow dominantly determine the alignment of CNTs.

References

1. M. Monfared, E. Shirani, F. Aloui and M. Salimpour, *Journal of Applied Fluid Mechanics*, 2016, **9**.
2. K. Kobashi, S. Ata, T. Yamada, D. N. Futaba, M. Yumura and K. Hata, *Chemical Science*, 2013, **4**, 727-733.
3. H. Yoon, M. Yamashita, S. Ata, D. N. Futaba, T. Yamada and K. Hata, *Scientific reports*, 2014, **4**, 1-8.
4. X. Chen, J. F. Dobson and C. L. Raston, *Chemical Communications*, 2012, **48**, 3703-3705.
5. T. Serra, J. Colomer and X. Casamitjana, *Journal of Colloid and Interface Science*, 1997, **187**, 466-473.
6. H. Cho, H. Lee, E. Oh, S.-H. Lee, J. Park, H. J. Park, S.-B. Yoon, C.-H. Lee, G.-H. Kwak, W. J. Lee, J. Kim, J. E. Kim and K.-H. Lee, *Carbon*, 2018, **136**, 409-416.
7. M. Ciszewski, D. Janas and K. K. Koziol, *Batteries*, 2019, **5**, 60.
